# LTF as a Potential Prognostic and Immunological Biomarker in Glioblastoma

**DOI:** 10.1007/s10528-024-10716-6

**Published:** 2024-05-19

**Authors:** Kai Qiu, Daling Ding, Fengjiang Zhang, Bo Yang

**Affiliations:** Department of Neurosurgery, The First Affiliated Hospital of Zhengzhou University, Zhengzhou, 450052 Henan Province China

**Keywords:** LTF, Glioblastoma, Survival, Immune cell infiltration

## Abstract

The lactoferrin (LTF) gene behaves like a tumor suppressor gene in diverse tumors, such as renal cancer, nasopharyngeal carcinoma and gastric cancer. However, the prognostic value of LTF expression in patients with glioblastoma remains unclear. In this study, the expression levels of LTF in patients with GBM were investigated in TCGA, GEPIA, CGGA and GEO database, and a survival analysis of LTF based on TCGA and CGGA was performed. Furthermore, the present study demonstrated the LTF gene co-expression, PPI network, KEGG/GO enrichment and immune cell infiltration analysis on TCGA and TIMER2.0 database. We found that LTF expression was significantly upregulated in GBM samples compared with normal samples and other glioma samples, and Kaplan–Meier analysis demonstrated that the overexpression of LTF were significantly associated with worse overall survival (OS) and 5-year OS in GBM patients (*P* < 0.05). KEGG/GO enrichment analysis demonstrated that functions of LTF concentrated in immune and inflammatory response and peptidase regulation (*P* < 0.05). Immune cell infiltration analysis presented that high LTF expression exhibited dysregulated immune infiltration (i.e., CD4 + T cells, neutrophils, macrophages, myeloid dendritic cells and cancer associated fibroblast). LTF was upregulated in tumors and correlated with worse OS in GBM patients, and LTF might function as an oncogene via inducing dysregulated immune infiltration in GBM.

**Supplementary Information:**

The online version contains supplementary material available at 10.1007/s10528-024-10716-6.

## Introduction

Glioblastoma multiforme (GBM) is the most aggressive and common primary brain tumour, which is classified as the highest-grade glioma by the World Health Organization (WHO). Currently, the mainstream therapeutic strategy for patients with GBM is surgical resection accompanied with radiotherapy and adjuvant chemotherapy. Despite these current treatments, including surgical resection combined with radiotherapy and temozolomide (TMZ) chemotherapy, its prognosis is still dismal deal to the development of resistance to TMZ over a period by GBM cells, the median survival of GBM patients is only about 12–15 months, and the 10-year survival rate in the cohort studied with GBM was estimated only 0.71%(Tykocki and Eltayeb 2018). Thus, finding new targets for the GBM treatment and efficient prognostic biomarkers is urgently required.

Lactoferrin (LTF) is a multifunctional protein distributing in many biological secretions including milk. It not only possesses iron binding/transferring but also demonstrates antibacterial, antiviral, and anti-inflammatory. Recently, an increased focus has been on elucidating the LTF in cancer, researchers have reported that LTF gene behaves like a tumor suppressor gene in diverse tumors, such as renal cancer, prostate cancer and gastric cancer et al.(Ni et al. 2020; Gołąbek et al. 2022; Zhao et al. 2021; Luo et al. 2015; Yi et al. 2006; Liu et al. 2022). They found that the absent expression or downregulation of LTF is widespread in these tumors and promotes tumor proliferation, while the overexpression of LTF inhibits the proliferation. Zhao et al. reported that overexpressed LTF–mediated JAK/STAT pathway to inhibit STAT3 expression and reduce tumor-derived GM-CSF secretion, regulate tumor immune microenvironment, and inhibit prostate cancer cell proliferation and migration. Low expression of LTF can promote the proliferation of prostate cancer (Zhao et al. 2021). Luo et al. reported that LTF expression was downregulated in gastric cancer and affected the MAPK signaling pathway (Luo et al. 2015). LTF might be a novel prognostic biomarker for these cancer types. However, there is a lack of reports on LTF in GBM and the expression level and prognostic value of LTF in GBM is still unclear. Therefore, to evaluate the prognostic value of LTF in GBM patients is necessary.

Here, we identified the LTF expression in GEO, GEPIA, CGGA and TCGA databases and performed a survival analysis based on CGGA and TCGA profile, in the hope of providing useful insights into GBM.

## Materials and Methods

### GBM Datasets and Preprocessing

Gene expression datasets of GBM were systematically searched from the Cancer Genome Atlas (TCGA; https://cancergenome.nih.gov/), Chinese Glioma Genome Atlas (CGGA; http://www.cgga.org.cn/) the Gene Expression Omnibus (GEO; https://www.ncbi.nlm.nih.gov/geo/).

In total, 5 GBM datasets containing tumor and normal samples were gathered, namely, TCGA-GBM (*n* = 173), GSE4290 (*n* = 180) (Fine HA et al., 2019), GSE116520 (*n* = 25) (Kruthika BS et al., 2018), GSE12657 (*n* = 25) (Moran LB et al., 2018), and CGGA mRNAseq_693 (all tumor, *n* = 693).

Count (RNA-sequence) data and clinical data of TCGA-GBM were obtained from the Genomic Data Commons (GDC) database. The raw microarray data from the GEO database was normalized by R software 4.3.1 with R package affy.

The pan-cancer analysis was processed by TIMER 2.0 database (http://timer.cistrome.org/) and GEPIA database (http://gepia.cancer-pku.cn/index.html).

The Single-Cell RNAseq analysis of GBM (GSE84465) was performed by R software 4.3.1 with R package Seurat.

### Survival Analysis

The LTF expression data and clinical data of the GBM patients from TCGA database were merged together by R software, and survival data of CGGA mRNAseq_693 was downloaded from CGGA. The overall survival (OS) and 5-year OS analysis was evaluated by Kaplan–Meier survival plot with GraphPad Prism 9.0 software.

### Gene Coexpression Analysis and Protein–Protein Interaction

The LTF coexpression analysis was performed by R software with limma package based on the TCGA-GBM dataset and visualized by Cytoscape software 3.8. The top 10 genes with Spearman-rank correlation > 0.6 were collected.

The protein–protein interaction (PPI) was analyzed by STRING database (https://cn.string-db.org/).

### KEGG/GO Enrichment

Kyoto Encyclopedia of Genes and Genomes (KEGG) and Gene ontology (GO) enrichment analysis were carried out via clusterProfiler package of R software (Yu et al. 2012). GO contained three categories: biological process, cellular component, and molecular function.

### Gene Mutation and Immune Infiltration Analysis

CBioPortal database (http://cbioportal.org) was adopted to analyze gene mutations of LTF in TCGA, CPTAC (Clinical Proteomic Tumor Analysis Consortium) and Columbia Nat Med GBM samples.

TIMER 2.0 database (http://timer.cistrome.org/) was adopted to analyze the associations between LTF expression and tumor-infiltrating immune cells infiltration levels.

### Statistical Analyses

Statistical analysis was conducted by the R software (version 4.03) and GraphPad Prism software (version 9.0). The Spearman correlation test was utilized for inferring the correlation between two parameters. *t* tests were adopted to compare the differences between two groups, while one-way ANOVA and Kruskal–Wallis tests were conducted to compare the differences between three or more groups. Two-tail *p*-values < 0.05 were considered statistically significant.

## Result

### Landscape of LTF Expression

**Fig. 1:**
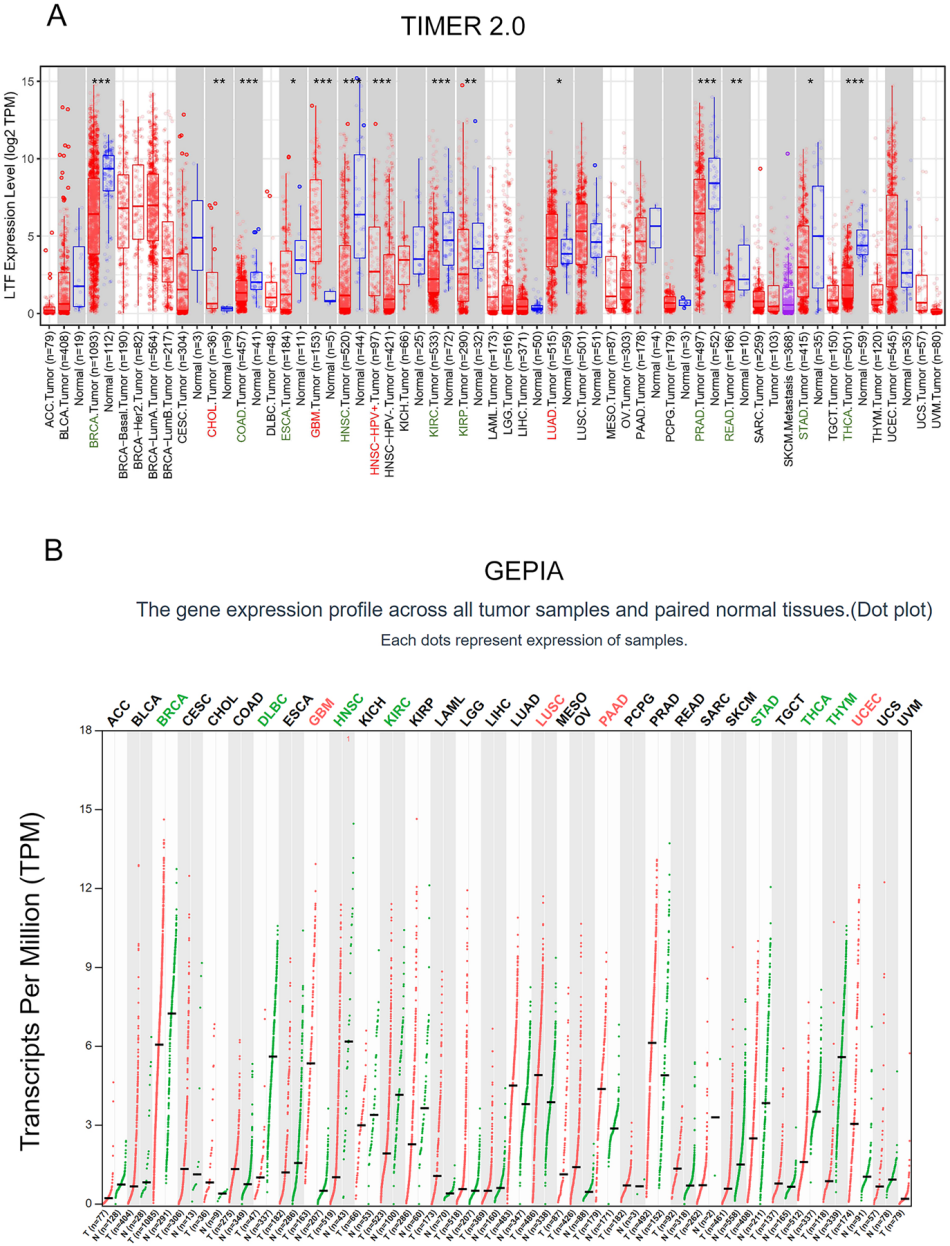
The expression level of LTF in all tumor tissues and cancer-side tissues based on TIMER 2.0 database(**A**) and GEPIA database(**B**) (*P < 0.05, **P < 0.01, ***P < 0. 001). The red cancer names denote the overexpression of LTF in this cancer, the green cancer names denote the low expression of LTF in this cancer, and black names represent no changes of LTF expression

In this investigation, we first explored the landscape of LTF expression in different cancers based on TIMER 2.0 and GEPIA database. The result showed that the expression of LTF was not significantly altered in most tumors included in TIMER (Fig. 1A, black names) and GEPIA (Fig. 1B, black names). Only in glioblastoma the level of LTF is significantly increased compared with normal samples in both TIMER and GEPIA (Fig. 1A, B red names), while the LTF expression is significantly decreased in breast cancer, gastric cancer and thyroid cancer (Fig. 1A, B green names).

**Fig. 2:**
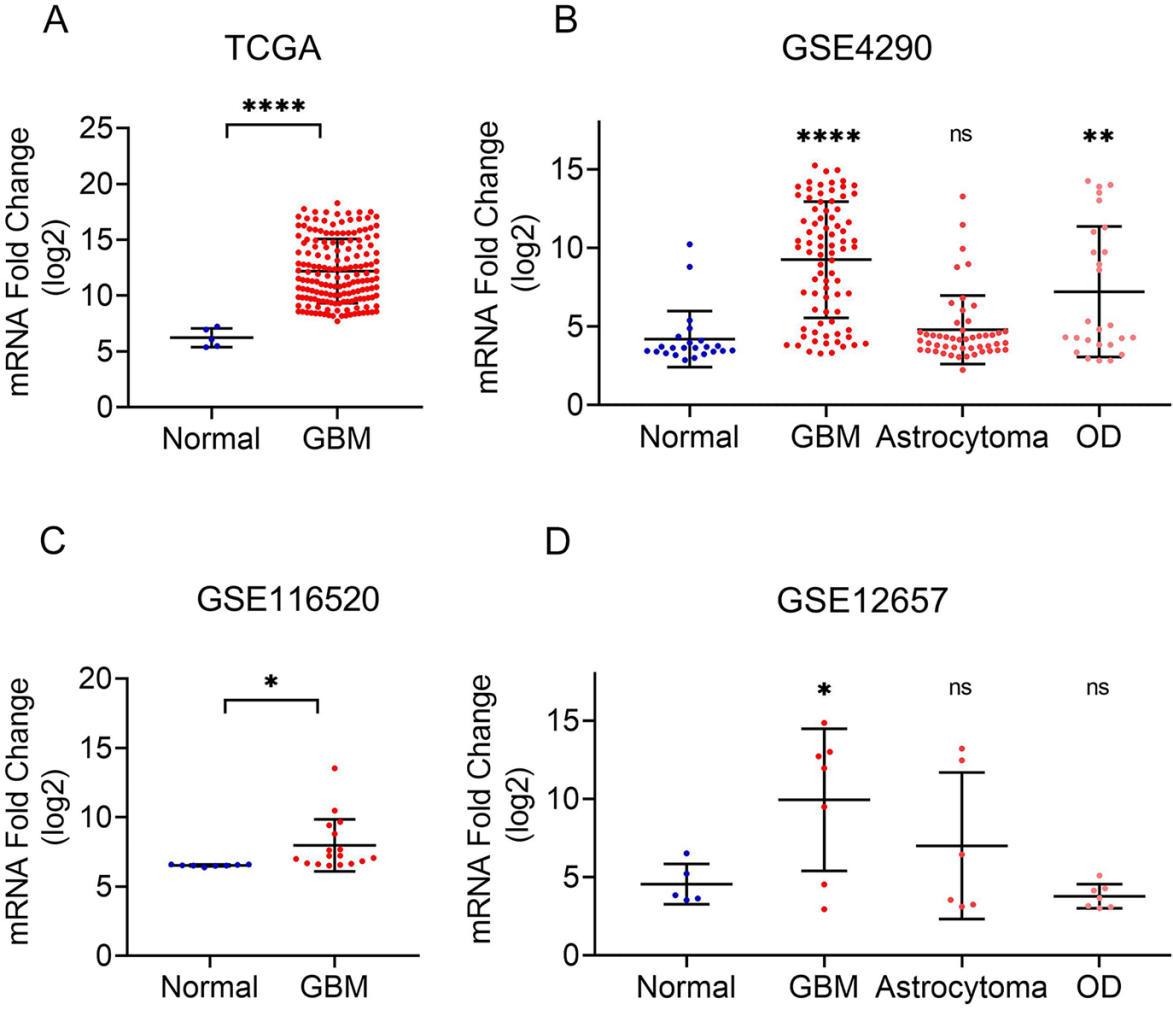
The comparison of LTF mRNA expression in normal and GBM samples base on TCGA and GEO database series including GSE4290(B), GSE116520(C) and GSE12657(D). ***p < 0.001, **p < 0.01, *p < 0.05 (*t*-test)

To further confirm the alteration of LTF in GBM, we next analyzed TCGA-GBM, GSE4290, GSE116520 and GSE12657 datasets (Fig. 2). The result showed that the expression level of LTF in GBM samples was significantly upregulated in all four datasets(*p* < 0.05). Together, these findings suggest that the mRNA level of LTF is significantly overexpressed in GBM.

Based on GSE4290 and GSE12657 datasets which also contained astrocytoma and oligodendroglioma samples, we next explored the LTF expression in these two low-degree gliomas. The result showed that there was a significant increase of LTF in oligodendroglioma compared with normal tissues in GSE4290 (Fig. 2B), while the LTF level was not significantly changed in astrocytoma in GSE4290 (Fig. 2B) and GSE12657 and oligodendroglioma in GSE12657 (Fig. 2D).

When compared with GBM tissues, the LTF level was significantly downregulated in astrocytoma(*p* < 0.0001) and oligodendroglioma(*p* = 0.0189) in GSE4290(Fig. 2B), and there was also a significant decrease of LTF in oligodendroglioma(*p* = 0.0041) in GSE12657(Fig. 2D), while no significance observed with astrocytoma in GSE12657 (*p* = 0.2773) (Fig. 2D).

### LTF Expression in Gliomas

**Fig. 3:**
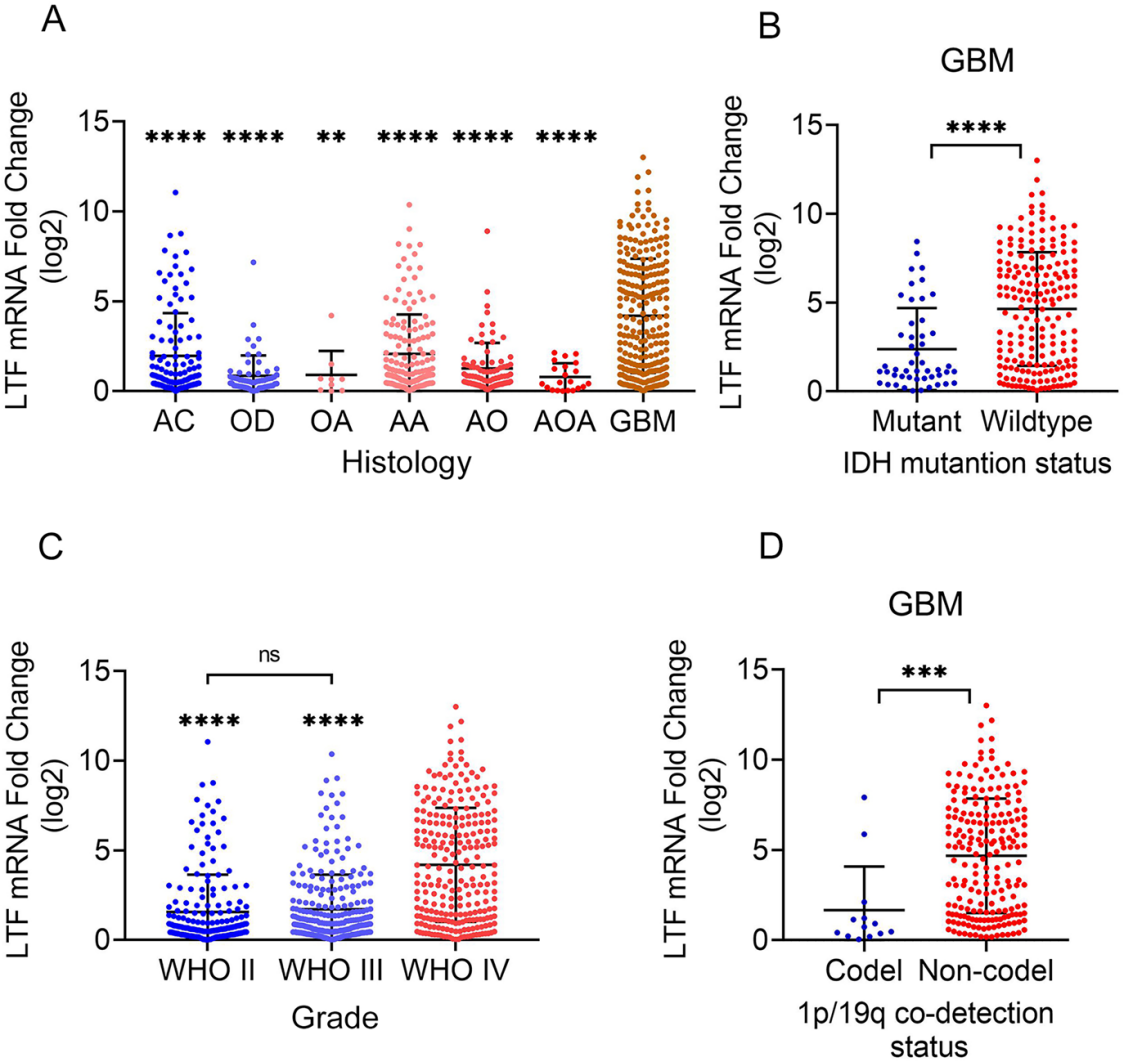
The comparison of LTF mRNA expression base on CGGA database grouped by histology of brain tumor(**A**), IDH mutation status(**B**), WHO grade of brain tumor(**C**) and 1p/19q co-detection status of GBM(D). ***p < 0.001, **p < 0.01, *p < 0.05 (*t*-test)

To further compare the LTF expression level of GBM with other low-degree gliomas, we utilized publicly available glioma data from CGGA. As shown in Fig. 3A, the LTF level of GBM was significantly higher than that was in astrocytoma (AC), oligodendroglioma (OD), oligoastrocytoma (OA), anaplastic astrocytoma (AA), anaplastic oligodendroglioma (OA) and anaplastic oligodendroid astrocytoma (AOA)(*p* < 0.05).

In addition, based on the CGGA mRNAseq_693 dataset, we further explored the LTF expression level in different clinical groups. As shown in Fig. 3C, compared with WHO IV grade glioma (GBM), LTF was significantly downregulated in lower grade gliomas (WHO II and WHO III). But there was no significance of LTF between WHO II and WHO III grade gliomas.

Isocitrate dehydrogenase (IDH) is a metabolic enzyme catalyzing the oxidative decarboxylation of isocitrate and plays key roles in the Krebs cycle and cellular homoeostasis(Han et al. 2020). IDH-mutation is clearly linked to the tumor genesis of human(Pirozzi and Yan 2021). We then analyzed the LTF expression level of GBM grouped by IDH mutation status, it showed that the LTF level was significantly increased in the wildtype group of GBM (Fig. 3B). Next, we compared the LTF level of GBM grouped by 1p/19q co-detection status, it showed that the LTF level was significantly upregulated in the non-codel group of GBM (Fig. 3D).

Taken together, the LTF expression level is significantly increased in GBM this highest WHO grade glioma.

### The LTF Expression in Single Cells Based on Single-Cell RNAseq Analysis

**Fig. 4:**
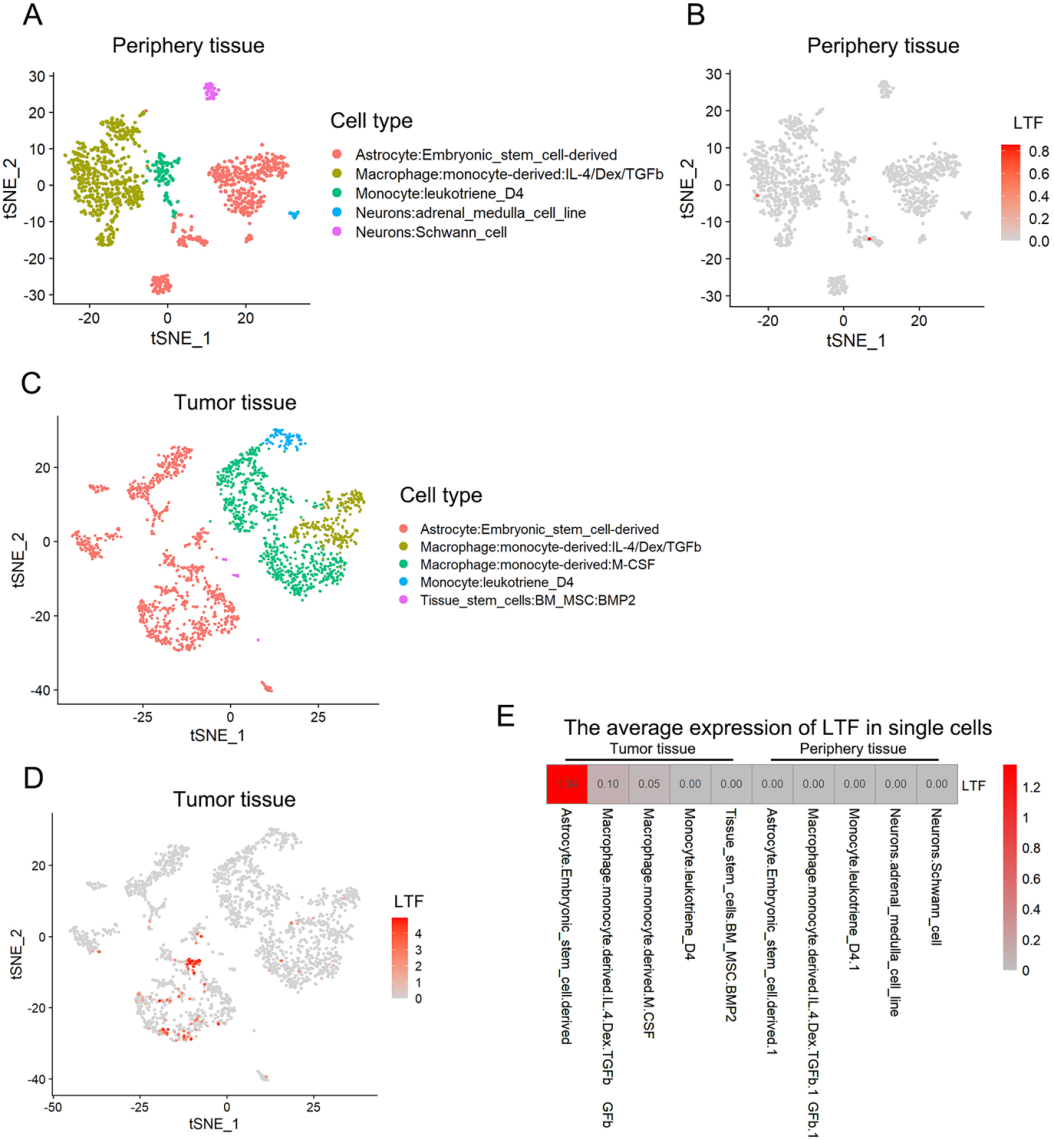
The LTF expression in single cells based on Single-Cell RNAseq analysis of diffuse neoplastic infiltrating cells in GBM (GSE84465). The Single-Cell RNAseq analysis of periphery tissue (**A**, **B**) and tumor tissue (**C**, **D**). The LTF expression level in different cells (**B**, **D**, **E**), red and grey colors denote the expression level of LTF high and low, respectively

We also performed Single-Cell RNAseq analysis to evaluate the LTF level in single cell–deprived from tumor tissue and periphery tissue of GBM. The t-stochastic neighbor embedding (t-SNE) plots showed that astrocytes, macrophages, monocytes and neurons were the main cells in periphery tissue (A), and astrocytes, macrophages, monocytes and tissue stem cells were the main cells in tumor tissue (C). And the expression of LTF was shown in Fig. 4B and D, red and grey colors denote the expression level of LTF high and low in single cells, respectively. The LTF kept at low level in all the cell types of periphery tissue and immune cells of tumor tissue, but obviously increased in tissue stem cells of tumor tissue. These results further validated the over expression of LTF in GBM, especially in tissue stem cells.

### Genomic mutation of LTF in GBM

**Fig. 5:**
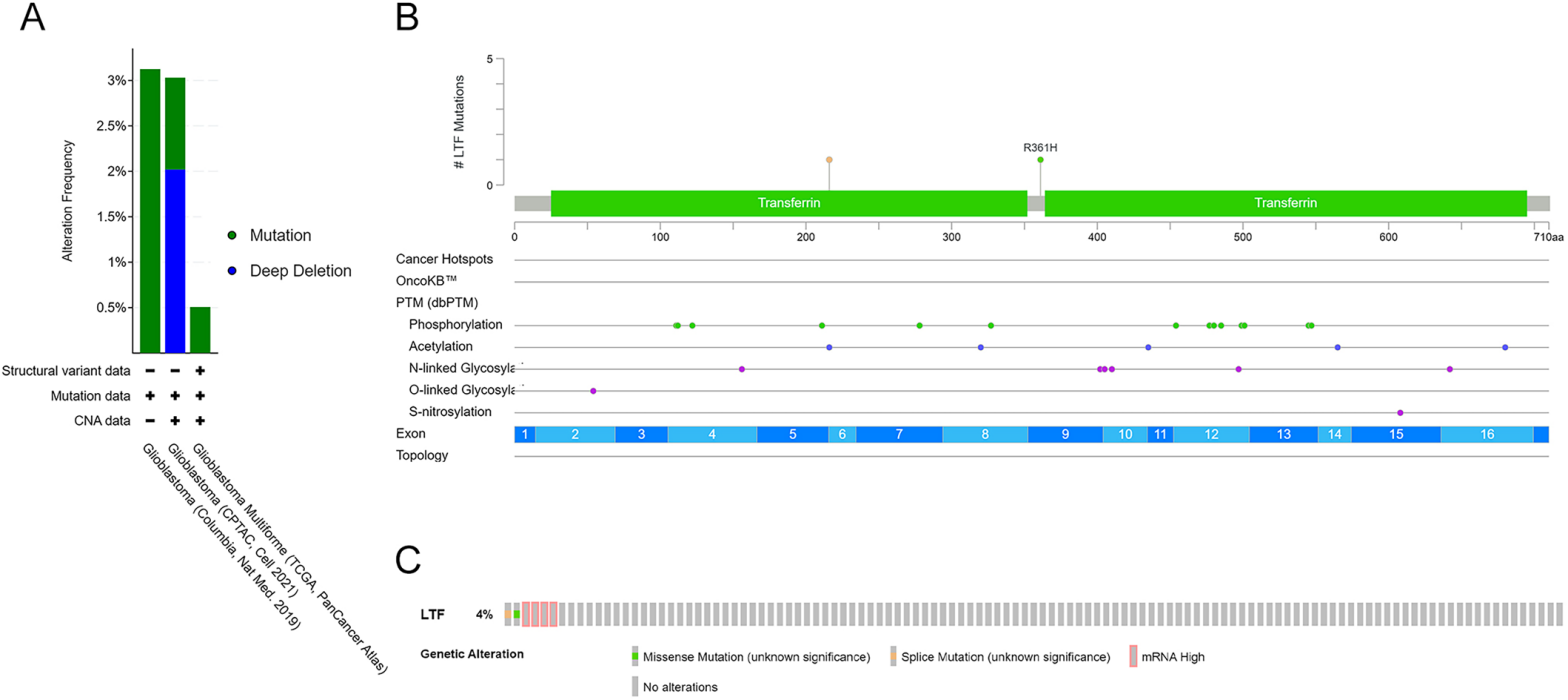
Mutation types and frequency of LTF in different data sets(**A**). Mutation sites of LTF(**B**). Mutation types and percentage of LTF in GBM (**C**)

We further analyzed the gene mutations of LTF on the cBioPortal database, and the results showed that the LTF mutation frequency was different in 3 datasets (Fig. 5 A), 3.13% (Columbia, Nat Med. 2019), 3.3% (CPTAC, Cell 2021) and 00.51% (TCGA, PanCancer Atlas) and there were 2 mutation sites in the LTF gene (Fig. 5B). Based on the TCGA PanCancer Atlas dataset, there was 1 case with LTF missense mutation, 1 case with splice mutation and 4 cases with mRNA high (Fig. 5C).

### The Prognosis of LTF in GBM Patients

**Fig. 6:**
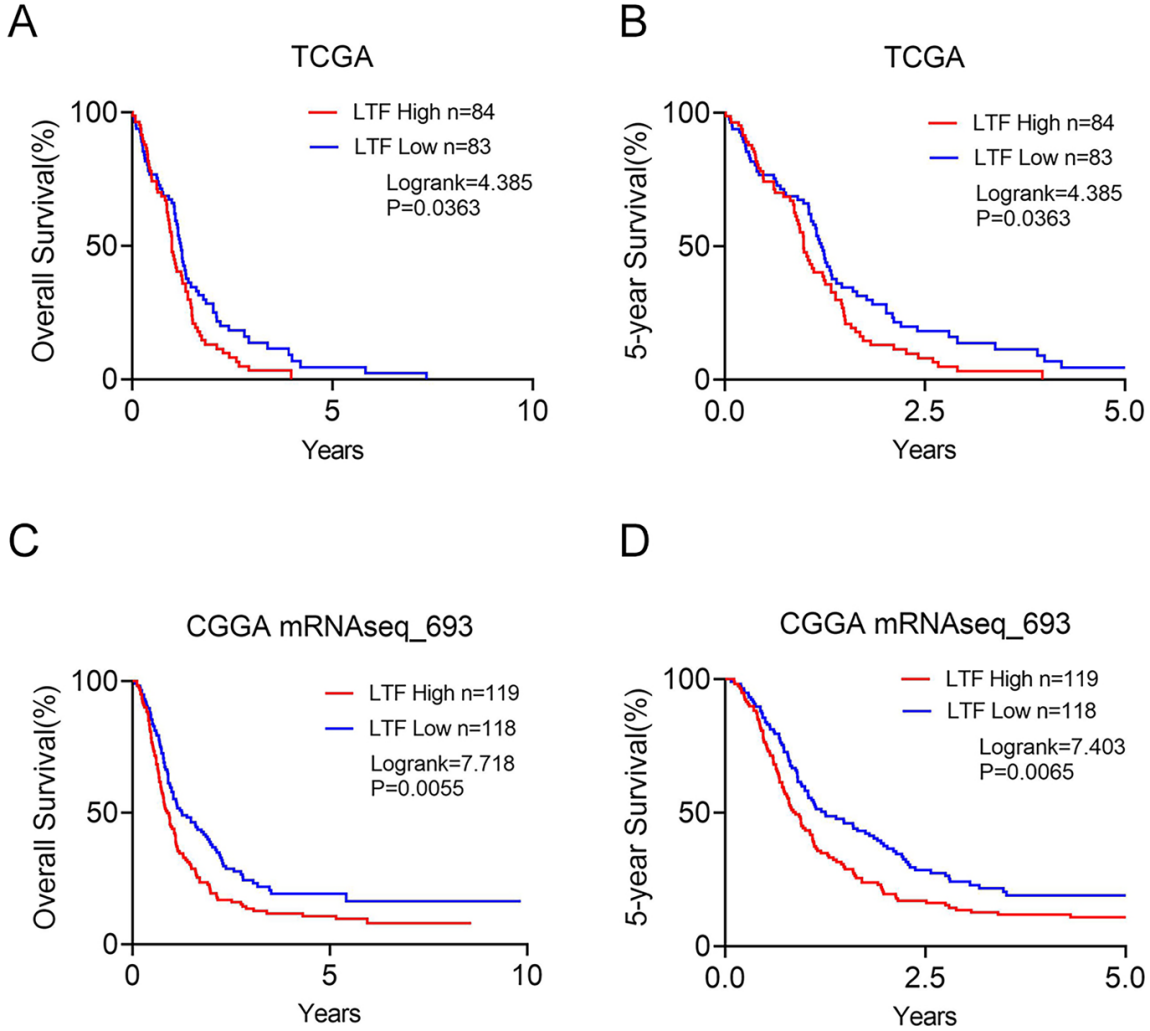
The overall survival (**A**, **C**) and 5-year overall survival (**B**, **D**) of GBM patients in TCGA and CGGA database grouped by the median of LTF expression

High-grade glioma is associated with dismal prognosis. Based on our results above, compared with WHO II-III grade glioma, LTF was significantly upregulated in higher grade gliomas (WHO IV), we hypothesized that the LTF expression level would correlate with the prognosis of glioma patients and GBM patients. Here, we performed Kaplan–Meier analysis of LTF and OS in glioma patients and GBM patients obtained from CGGA mRNA_693 and TCGA-GBM datasets, which showed that the LTF overexpression in tumor tissues was significantly associated with poor OS and 5-year OS in glioma patients (Log-rank *p* < 0.05, Fig. S1A, B) and GBM patients (Log-rank *p* < 0.05, Fig. 6A–D).

### The Association Between LTF and Clinical Characteristics of GBM Patients

As shown in Table 1, There were more patients with age > 50, IDH wildtype and x1p19q non-codel (*p* < 0.05) in LTF high group. There was no significant difference in gender and primary GBM or not between the two groups (*p* > 0.1).

**Table 1:** Characteristics of GBM patients between LTF high and low groups

Variables		LTF expression level		*P* value
		High n = 124	Low n = 125	
Age, years (%)	≤ 50	55 (44.4)	75 (60.0)	0.019
	> 50	69 (55.6)	50 (40.0)	
Gender (%)	Female	45 (36.3)	57 (45.6)	0.172
	Male	79 (63.7)	68 (54.4)	
GBM type (%)	Primary	65 (52.4)	75 (60.0)	0.281
	Recurrent	59 (47.6)	50 (40.0)	
IDH mutation status (%)	Mutant	12 (10.1)	37 (30.8)	< 0.001
	Wildtype	107 (89.9)	83 (69.2)	
X1p19q codel status (%)	Codel	2 (1.7)	11 (10.9)	0.01
	Non-codel	115 (98.3)	90 (89.1)	

### LTF Coexpression and PPI

**Fig. 7:**
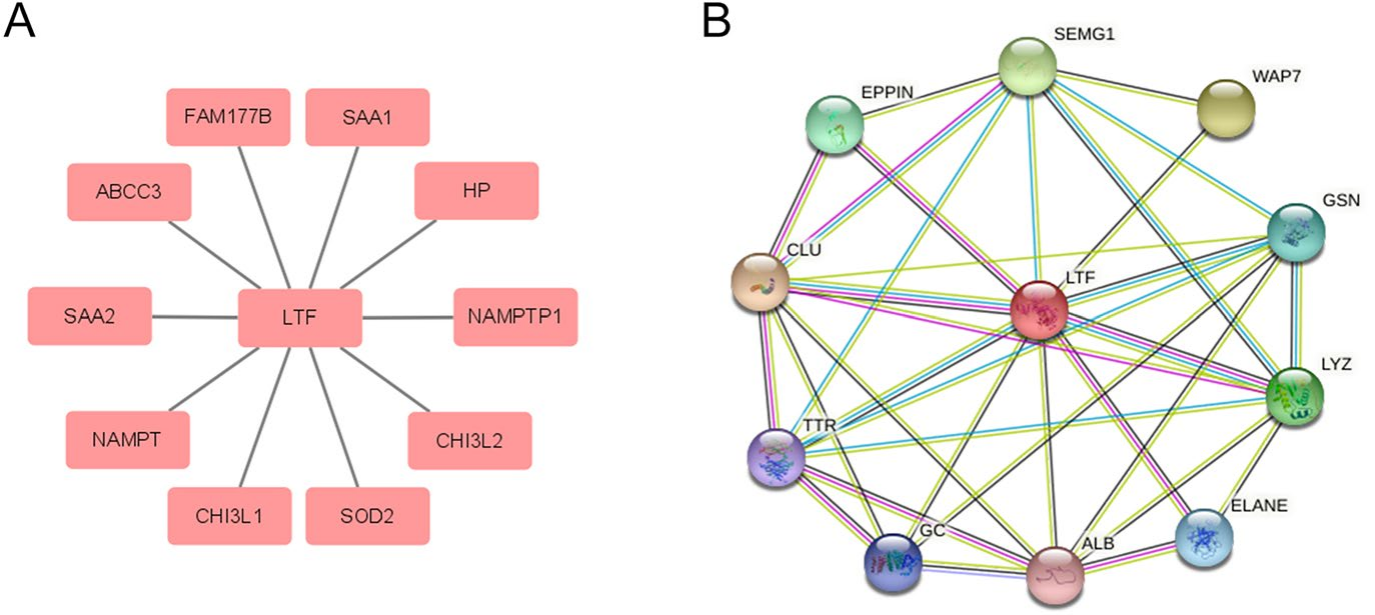
**A** The top 10 most closely coexpressed genes of LTF based on TCGA-GBM dataset. **B** The PPI network of LTF in STRING

We further explored the gene co-expression of LTF in GBM patients based on the TCGA-GBM dataset. The top 10 co-expression genes with Spearman correlation > 0.6 including FAM177B, SAA2, HP, NAMPT, NAMPTP1, CHI3L2, CHI3L1, SAA1, SOD2 and ABCC3 were obtained as shown in Fig. 7A.

We next investigated the PPI network with STRING, which revealed that 10 genes including SEMG1, EPPIN, CLU, TTR, GC, ALB, ELANE, LYZ, GSN and WAP7 were interacting with LTF (Fig. 7B).

### GO/KEGG Biological Process Enrichment

**Fig. 8:**
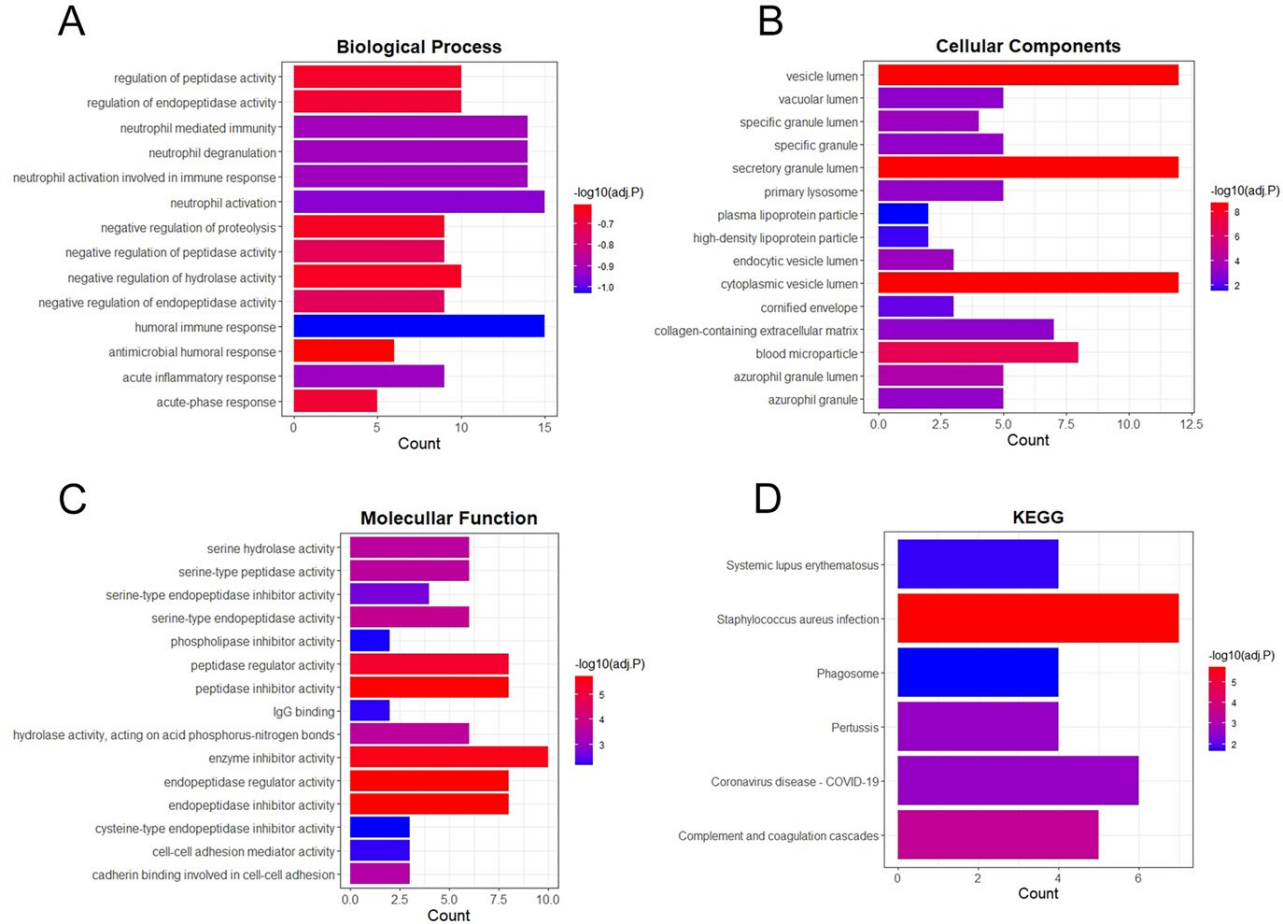
GO and KEGG biological process enrichment of LTF interactive genes. Biological process(**A**), Cellular components(**B**), Molecular function(**C**) and KEGG(**D**)

GO analysis resulted that neutrophil activation and humoral immune response of biological process, secretory granule lumen and vesicle lumen of cellular components and enzyme inhibitor activity and peptidase regulator activity of molecular function were the most enriched function of LTF interactive genes (Fig. 8A–C). The KEGG enrichment of LTF interactive genes showed that Staphylococcus aureus infection and Coronavirus disease—COVID-19 were the most enriched pathway (Fig. 8D).

### The Association Between Immune Infiltrates and LTF in GBM

**Fig. 9:**
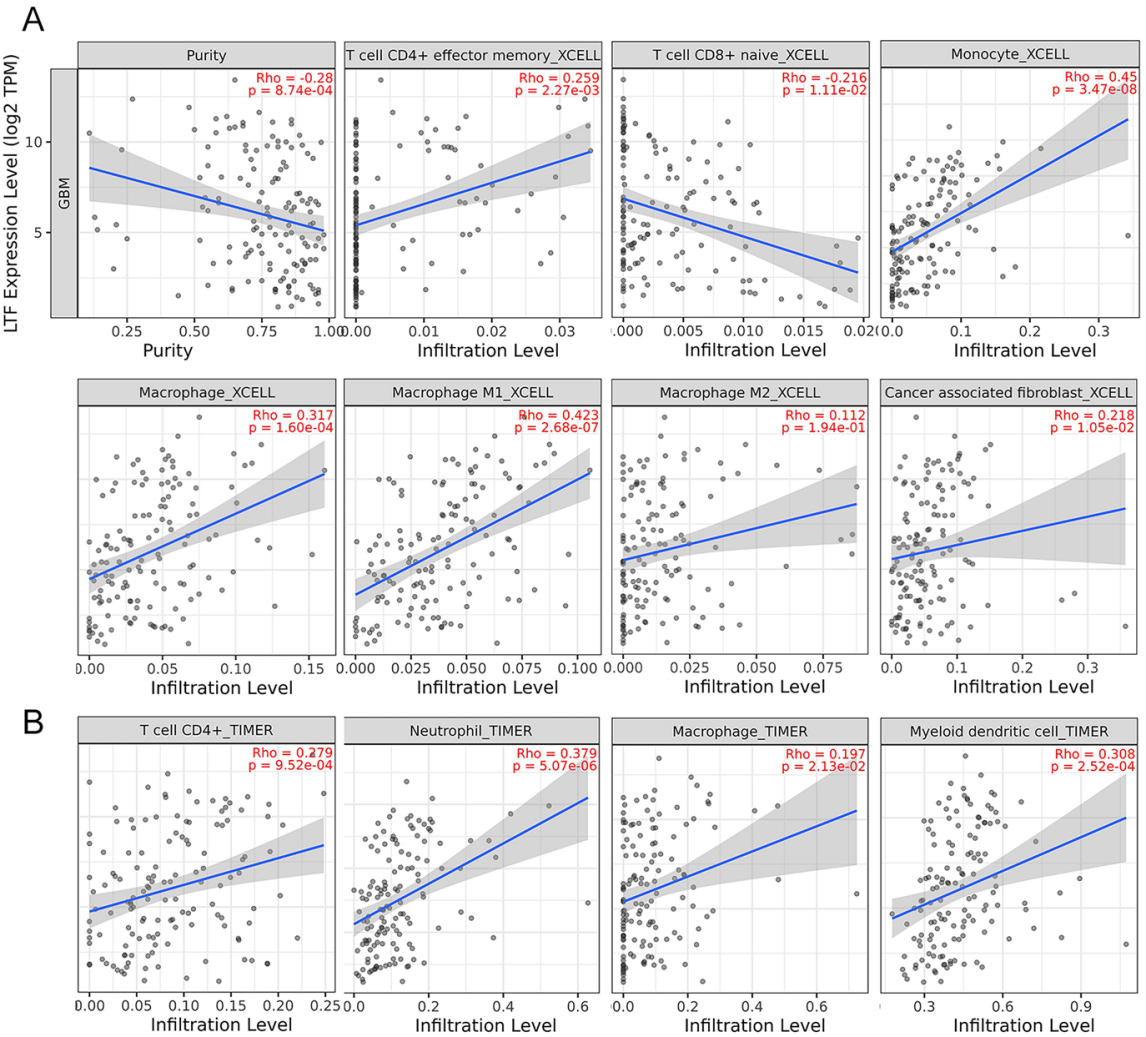
Correlation between LTF expression level and GBM immune cells infiltration level

Based on TIMER 2.0 database, we next uncovered that LTF expression was negatively correlated with tumor purity (Rho = − 0.28, *p* = 8.74e-04). We further used XCELL algorithm and found that LTF expression was significantly correlated to the infiltration of different immune cells, including CD4 + effector memory T cells (Rho = 0.259, *p* = 2.27e-03), CD8 + naive T cells (Rho = − 0.216, *p* = 1.11e-02), monocytes (Rho = 0.45, *p* = 3.47e-08), macrophages (Rho = 0.317, *p* = 1.60e-04) and cancer associated fibroblast (Rho = 0.325, *p* = 6.40e-10) (Fig. 9A). We also explored the immune infiltrations with TIMER algorithm and found that LTF expression was also significantly correlated to the infiltration of neutrophils (Rho = 0.379, *p* = 5.07e-06) and myeloid dendritic cells (Rho = 0.308, *p* = 2.52e-04) (Fig. 9 B).

## Discussion

In the present study, we demonstrated that LTF was upregulated in GBM samples, which associated with worse overall survival in GBM patients. In addition, this adverse effect of high LTF expression on the prognosis of GBM patients might be due to the correlation with multiple oncogenes and immune infiltrations.

Lactoferrin (LTF) is a multifunctional protein secreted in many tissue fluids including tears, saliva and milk. It provides protective effects such as antibacterial, antiviral, antifungal, anti-inflammatory and anti-carcinogenic properties(Rosa et al. 2017). Recently, several studies have reported that LTF was downregulated and may act as a tumor suppressor in several types of cancer such as prostate cancer, clear cell renal cell carcinoma, oral squamous cell carcinoma and nasopharyngeal carcinoma.

Previous studies found that LTF was kept at a low level in tumor tissue and artificially upregulated the LTF expression in tumor cells could lead to growth suppression in vitro, which means LTF was acting as a tumor suppressor in those cancers. Ni et al. found that LTF significantly down regulated in renal cancer, and overexpressed LTF could suppress the capacity of migration and invasion in clear cell renal cell carcinoma cell lines (Ni et al. 2020). Zhao et al. reported that they overexpressed the LTF gene in the prostate adenocarcinoma PC-3 cell line and found high LTF indirectly interfere with the local immunosuppressive function of myeloid-derived suppressor cells to inhibiting tumor cell proliferation and migration (Zhao et al. 2021).Interestingly, in our study we found that LTF was significantly overexpressed in glioma blastoma, and the LTF level in GBM was also significantly higher than that was in other low WHO grade gliomas such as astrocytoma, oligodendroglioma and oligoastrocytoma et al. In addition, Single-Cell RNAseq analysis further validated the over expression of LTF in GBM, especially in tissue stem cells. This result suggests that LTF may be correlated with the tumor malignancy and clinical outcomes of GBM.

To test this hypothesis, we further performed Kaplan–Meier survival analysis based on the clinical data of TCGA and CGGA datasets. The Kaplan–Meier plot resulted that the GBM patients with high LTF expression exhibited poor prognosis. LTF overexpression in GBM patients was associated with poor overall survival and 5-year overall survival. Taken together, the LTF was overexpressed and may not act as a tumor suppressor in GBM, which was quite different from that in other tumors.

Thus, we next explored the gene co-expression and function of LTF in GBM based on TCGA GBM datasets. The results showed that the top 10 co-expression genes were FAM177B, SAA2, HP, NAMPT, NAMPTP1, CHI3L2, CHI3L1, SAA1, SOD2 and ABCC3. Among which, SAA1, SAA2(Rosa et al. 2017; Sun and Ye 2016; Takehara et al. 2020; Zhang et al. 2021), HP(Oh et al. 2023), NAMPT(Bi and Che 2010; Gasparrini and Audrito 2022; Wei et al. 2022), CHI3L1 and CHI3L2 (Libreros et al. 2013; Pusztai et al. 2019) have been reported to act as tumor promoters in multiple cancers including lung cancer, breast cancer and ovarian cancer. NAMPT also has become a therapeutic target for cancers. These promising genes may help us to understand the function of LTF in GBM. Moreover, the PPI network analysis showed that LTF was interacting with 10 genes including SEMG1, EPPIN, CLU, TTR, GC, ALB, ELANE, LYZ, GSN and WAP7. SEMG1(Shuvalov et al. 2020), CLU(Zhang et al. 2023) and GSN(Feldt et al. 2019) have been reported to be associated with cancers.

We further analyzed the GO/KEGG biological process of LTF, the results showed that LTF also played an important role in immune responses, enzyme inhibitor activity and infection, which suggested that the LTF expression of GBM may be associated with immune infiltrates. Then we performed the analysis of immune infiltrates on TIMER 2.0 database, the results showed that the LTF expression in GBM was negatively correlated with tumor purity and positively correlated with CD4 + effector memory T cells, macrophages, neutrophils and cancer associated fibroblast, suggesting the LTF may also indirectly regulate GBM by immune infiltrates.

In conclusion, our results demonstrated that LTF expression was upregulated in GBM, and its overexpression might cause worse overall survival in GBM patients. Further, the adverse effect of high LTF expression on the survival outcome of GBM patients might be due to the correlation with multiple oncogenes and immune infiltrates. Our findings may provide new insights into GBM prognosis and pathogenesis.

## Supplementary Information

Below is the link to the electronic supplementary material.

Supplementary file1 (PDF 13 KB)
