# Supplementary material for: LTF as a Potential Prognostic and Immunological Biomarker in Glioblastoma
Source: Biochem Genet. 2024 May 19;63(3):2347–62. doi: 10.1007/s10528-024-10716-6 (PMC12143989; doi:10.1007/s10528-024-10716-6)
Supplement: Supplementary file 1 — Supplementary file1 (PDF 13 KB) [file 10528_2024_10716_MOESM1_ESM.pdf]

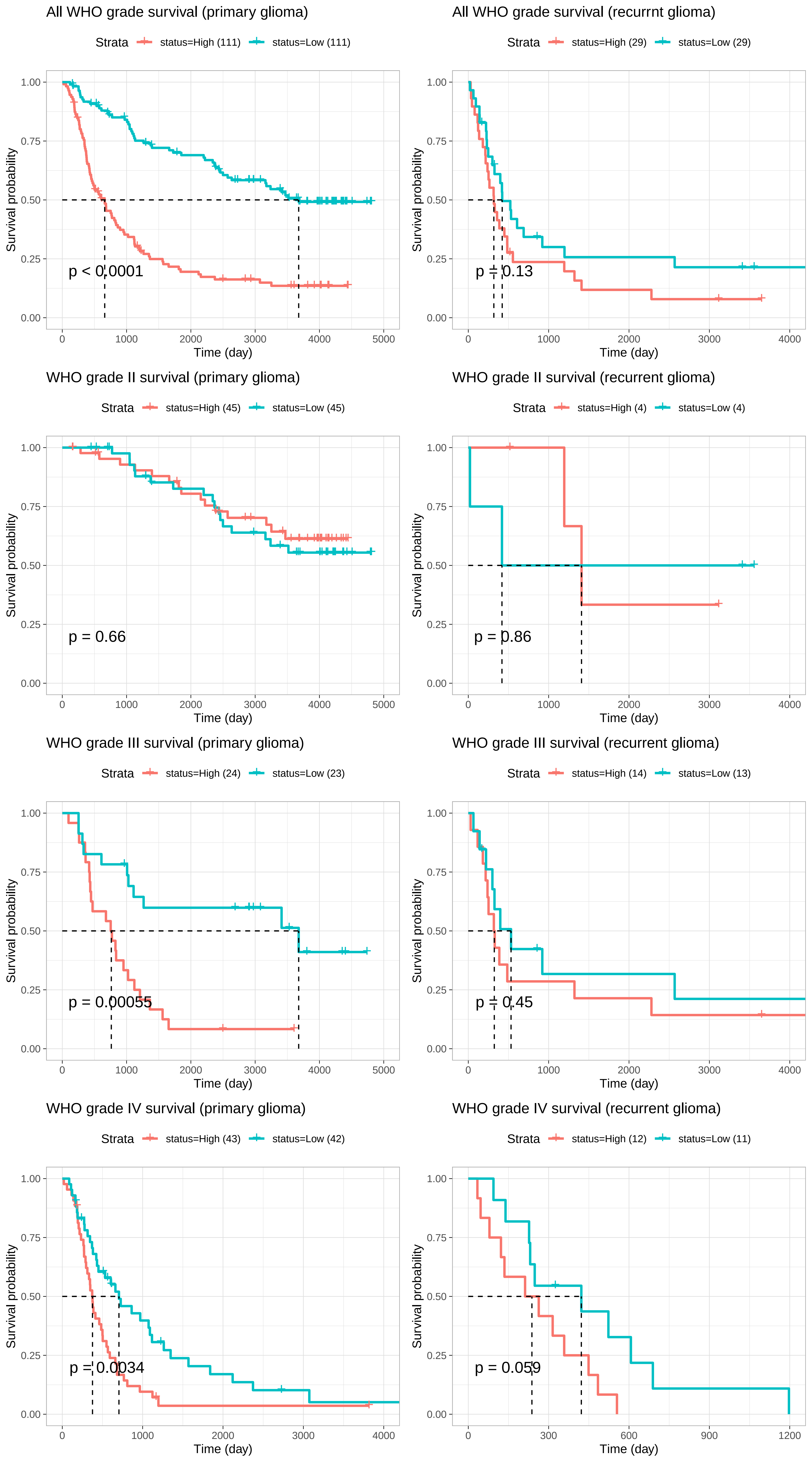
All WHO grade survival (primary glioma)
Strata status=High (111) status=Low (111)
Survival probability
1.00
0.75
0.50
0.25
0.00
p < 0.0001
0
1000
2000
3000
4000
5000
Time (day)
All WHO grade survival (recurrnt glioma)
Strata status=High (29) status=Low (29)
Survival probability
p = 0.13
0
1000
2000
3000
4000
Time (day)
WHO grade II survival (primary glioma)
Strata status=High (45) status=Low (45)
Survival probability
p = 0.66
Time (day)
WHO grade II survival (recurrent glioma)
Strata status=High (4) status=Low (4)
Survival probability
p = 0.86
Time (day)
WHO grade III survival (primary glioma)
Strata status=High (24) status=Low (23)
Survival probability
p = 0.00055
Time (day)
WHO grade III survival (recurrent glioma)
Strata status=High (14) status=Low (13)
Survival probability
p = 0.45
Time (day)
WHO grade IV survival (primary glioma)
Strata status=High (43) status=Low (42)
Survival probability
p = 0.0034
0
1000
2000
3000
4000
Time (day)
WHO grade IV survival (recurrent glioma)
Strata status=High (12) status=Low (11)
Survival probability
p = 0.059
0
300
600
900
1200
Time (day)
